# Femtosecond Laser Direct Writing of Flexible Electronic Devices: A Mini Review

**DOI:** 10.3390/ma17030557

**Published:** 2024-01-24

**Authors:** Shutong Wang, Junjie Yang, Guoliang Deng, Shouhuan Zhou

**Affiliations:** 1 College of Electronics and Information Engineering, Sichuan University, Chengdu 610064, China; wangst@scu.edu.cn (S.W.);; 2 North China Research Institute of Electro-Optics, Beijing 100015, China

**Keywords:** femtosecond laser direct writing, physical mechanisms and characteristics, spatial–temporal pulse shaping, flexible supercapacitor, triboelectric nanogenerator, wearable sensors, flexible optoelectronics

## Abstract

By virtue of its narrow pulse width and high peak power, the femtosecond pulsed laser can achieve high-precision material modification, material additive or subtractive, and other forms of processing. With additional good material adaptability and process compatibility, femtosecond laser-induced application has achieved significant progress in flexible electronics in recent years. These advancements in the femtosecond laser fabrication of flexible electronic devices are comprehensively summarized here. This review first briefly introduces the physical mechanism and characteristics of the femtosecond laser fabrication of various electronic microdevices. It then focuses on effective methods of improving processing efficiency, resolution, and size. It further highlights the typical progress of applications, including flexible energy storage devices, nanogenerators, flexible sensors, and detectors, etc. Finally, it discusses the development tendency of ultrashort pulse laser processing. This review should facilitate the precision manufacturing of flexible electronics using a femtosecond laser.

## 1. Introduction

The fast advancements in optics, nano photonics, optoelectronics, and biomedical engineering have placed increased demand on the manufacturing of micro/nanodevices. As a novel production tool, femtosecond laser technology offers the benefits of high accuracy, flexibility, mask-free processing, and the ability to process a variety of materials. Recently, an increasing number of studies have focused on the micromachining of micro/nanostructure, optical waveguides, and gratings, which have significantly promoted the development and application of flexible supercapacitors, triboelectric nanogenerators, press/stress sensors, photoelectronic detectors, and other devices. Various functional materials and femtosecond laser processing techniques applied to flexible electronic devices have been extensively studied. In order to realize multifunctional integration, it is often necessary to introduce more specific materials, as well as complex processing techniques. Among the many functional materials, metal-based materials, carbon-based materials, and chalcogenides have attracted much attention. However, due to the different material properties, the laser processing treatment processes involved are slightly different, such as laser reduction, laser sintering, laser carbonization, etc. To improve the precision and efficiency of laser processing, different processing means have also been adopted, such as dot direct writing, array direct writing, or exposure. Although there have been research review articles on femtosecond laser processing and applications in recent years, more attention has been paid to fields like photonic devices, micro-optical components, and functional surfaces, etc. [1,2,3] There are also a few articles that summarize research efforts in flexible electronics, but they lack integration in terms of processing mechanisms and methods [4]. Therefore, we summarize the recent progress in femtosecond laser processing of several functional materials and methods that are being developed in the field of flexible electronics. The purpose of this mini review is not to cover the entire field of femtosecond laser processing, which was the subject of recent a review paper [5], but to summarize its progress. Indeed, since the results discussed in the previous review paper [4,6], a large number of new contributions have been made to the field by femtosecond laser processing technology. Due to the vast amount of literature in the field over the past decades, this mini review attempts to provide an in-depth overview of representative work in each topic area without losing the breadth of scope.

This mini review focuses on the latest advancements in femtosecond laser micromachining that particularly emphasizes the applications of flexible electronic devices. The paper is organized as follows. Section 2 introduces the physical mechanisms and characteristics of femtosecond laser processes and compares them with long pulse processes. Section 3 illustrates the femtosecond laser fabrication methods to improve the processing efficiency and processing technology on different kinds of flexible electronics materials, like metal nanoparticles, metal oxides nanoparticles, graphene oxide materials, and some polymer materials. Section 4 reviews various flexible electronic devices processed by femtosecond laser and the corresponding applications, including flexible energy storage device, triboelectric nanogenerator, flexible sensor, and flexible detector. Finally, Section 5 discusses the prevailing challenges and future prospects in relevant fields.

## 2. Physical Mechanisms and Characteristics of Femtosecond Laser Processing

Femtosecond laser–material interaction is a process across multiple time scales and multiple space scales, involving a large number of complex physical and energy coupling mechanisms, such as laser energy absorption, electron excitation, electron heat diffusion, electron–lattice energy exchange, surface electron emission, ionization, plasma generation, and expansion, etc. The interaction processes with femtosecond laser vary greatly for different types of materials due to their different electron energy level structure and lattice structure, as well as nonlinear properties under different laser fluences [7,8]. Reference [9] gives a summarized distribution in time of the physical processes resulting from the interaction of femtosecond laser pulses with materials. In the initial stage, the absorption of photon energy by electrons is the primary process, which takes place on the femtosecond time scale after irradiation of the material with a femtosecond laser. The main mechanism of free electron excitation is strong electric field ionization (multi-photon ionization and tunneling ionization). Subsequently, on the picosecond time scale after femtosecond laser irradiation of the material, energy transfer with chemical bond breaking occurs, i.e., processes such as lattice heating and phase transitions. Finally, on the nanosecond or longer timescale, there is relaxation and recombination at the material surface, corresponding to phenomena such as plasma expansion, radiation, and matter jets. Therefore, when a femtosecond laser irradiates material, the laser deposits energy into the material and transfers it to the electrons, which transfer it to the atoms and lattice, causing the lattice to heat and react, ultimately resulting in the structure and phase transition, and/or ablating the material.

The specific mechanisms followed by femtosecond laser interaction with different materials vary somewhat due to the different properties of the materials, such as their electronic energy level structure and lattice structure. Typically, there are many free electrons within metallic materials. When a femtosecond laser irradiates a metal, the free electrons in the conduction band jump to higher energy levels mainly by absorbing photon energy through inverse bremsstrahlung [10,11]. The energy is distributed to the free electrons by electron–electron collisions, and the free electron system forms a non-equilibrium state. Due to an imbalance and energy relaxation between the electron and lattice systems, the femtosecond laser gradually heats up the lattice through an electron–phonon scattering and energy coupling process. Since the phonon mass is much larger than the electron mass and the time for the electron to transfer energy to the phonon is much longer than the laser pulse duration, the lattice temperature is usually assumed to be essentially unchanged during the irradiation time of a single femtosecond laser pulse [12]. After completing the ultrafast pulsed irradiation, the lattice is heated to a critical temperature. This leads to critical point melting [13], cleavage [13,14], phase separation [15], and finally, phase explosion ablation [16]. In contrast, nonmetallic materials, like semiconductor and dielectric materials, lack a significant number of free electrons, which are primarily excited through single-photon ionization, multi-photon ionization, and tunneling ionization [17,18]. The valence band electrons then gain leaps through collisional ionization, leading to a rapid increase in the free electron density and a rapid increase in temperature, followed by energy transfer to the lattice and a thermal or non-thermal phase transition of the material [19,20,21]. Thermal phase changes mainly include melting, vaporization, phase explosion, fragmentation, and cracking. During the non-thermal phase transition process, low laser energy may cause the crystalline state of the material to shift from crystalline to amorphous or single crystal to polycrystalline [22]. As laser energy increases, high-energy electrons will escape, leaving behind positive ions. The Coulomb force between these positive ions causes them to repel each other, leading to an explosive removal of material known as Coulomb explosion [23,24]. Depending on material properties and parameters, such as laser fluence, different phase transition mechanisms may coexist and transform [9,25,26].

**Figure 1:**
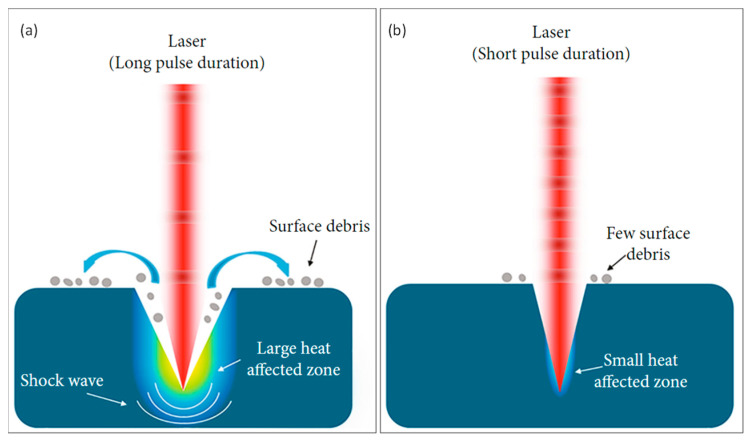
Illustration of laser–matter interaction characteristics: (**a**) long pulse and (**b**) short pulse(the gradient color represents the heat affected zones). (Adapted from [1] on the basis of open access).

Laser processing technology changes the state and properties of the material through the interaction of the laser with the material, thus realizing structure, phase, and property control at different scales. The significant differences between long pulse and femtosecond laser pulse micromachining are depicted in Figure 1. In the case of long pulses (e.g., >1 ns), the material has time to heat up, melt in the focal volume, and diffuse into the surrounding material. This results in the vaporization and ejection of some of the molten material in the form of high-velocity micro-droplets. However, the remaining melt re-solidifies, resulting in suboptimal machining quality manifested as heat-affected zones, recasts, slag, and micro-cracks. For ultra-short pulses (e.g., <1 ps), the thermal diffusion length is generally much smaller than the light penetration length [24,27]. This results in rapid ionization and the direct conversion of the material within the irradiated volume into a mixture of plasma, vapors, and nanodroplets, which are then ejected. Since minimal heat diffused into the surrounding area, the process yields high-quality outcomes that are clean and precise, without any of the defects commonly associated with longer pulse durations.

Femtosecond laser processing differs from longer pulse processing in three ways: (1) Cold processing. The extended pulse duration of long-pulsed lasers leads to a substantial thermal effect during material processing, reducing processing precision. The pulse duration of femtosecond lasers is usually in the tens to hundreds of femtoseconds range, which may fundamentally alter the mechanism of action when interacting with the material, resulting in high-precision processing. The carriers are excited by absorbing photon energy within an extremely short period during femtosecond laser irradiation, while the material’s lattice remains essentially unchanged. After the laser pulse ends, electron–lattice scattering enables energy transfer from the electrons to the lattice. Therefore, the thermal diffusion around the laser action area is negligible when the laser pulse width is smaller than the electron–phonon coupling time. The characteristic of femtosecond lasers, known as “cold processing”, is highly significant in micro and nanofabrication, as it effectively reduces thermal diffusion while processing. (2) Various types of materials can be treated [20,24]. Femtosecond lasers are known for their incredibly high peak power, which can easily exceed 10^12^ W/cm^2^. Due to the extremely high intensity, nonlinear mechanisms, including avalanche ionization, multi-photon absorption, and Coulomb explosion reactions, occur when the femtosecond laser interacts with the material [28,29]. Most materials, such as metals, polymers, semiconductors, and transparent dielectrics, can be processed using femtosecond lasers due to the high laser intensity exceeding the photoexcitation threshold of these materials, leading to light absorption and completion of the process. (3) High-resolution processing to exceed the diffraction limit ~λ/2, where λ is the light wavelength. The multi-photon absorption effect of the material can be brought about by the femtosecond laser’s extremely high-power density, and the electrons can be excited by absorbing multiple photons simultaneously. Typically, the laser intensity of the femtosecond laser is Gaussian distributed in space, and its peak intensity is only sufficient to bring about multi-photon absorption and thus realize the interaction with the material when it is close to the focal point of focus. In contrast, long pulses or continuous lasers can only interact with material through single-photon excitation at a wide range of laser intensity, which requires photon energy greater than the material’s band gap. As a result, light with photon energy below the band gap cannot directly excite the electron unless the laser intensity is high enough to trigger a nonlinear multiphoton excitation. The processing accuracy is low because the single photon’s energy absorption pattern matches the focused spot’s pattern spatially, and the thermal diffusion depth is much larger than the light wavelength [27].

As industries like advanced communications, green energy, and biotechnology demand devices that are smaller, more integrated and more responsive, advanced technologies are needed to support their rapid development. Currently, micro and nanofabrication technologies are primarily based on silicon-based processing. However, with the increasing diversity of micro and nanotechnology applications, the limitations of silicon-based processing are becoming more apparent. Femtosecond laser microfabrication technology is becoming a precise processing tool for micro and nano manipulation of materials due to its unique characteristics. This makes it widely applicable to the preparation of flexible electronic devices.

## 3. Practical Processing Technologies Using Femtosecond Laser

Femtosecond direct writing processing technology is more flexible and has a high degree of freedom, and is applicable to a variety of point, line, and layer processing. It is also widely integrated with spatial–temporal pulse shaping technology beam projection and/or interference transformation to prepare micro-nanostructures to improve the processing accuracy and efficiency. Based on the effects of femtosecond laser, laser processing can be applied for innovative mechanisms, such as the sintering of nanometallic materials, the reduction of metal oxides or graphene oxide materials, laser nanojoining, and the carbonization of some polymer materials. Their unique technical features and typical research are then reviewed in this section.

### 3.1. Direct Femtosecond Laser Writing

**Figure 2:**
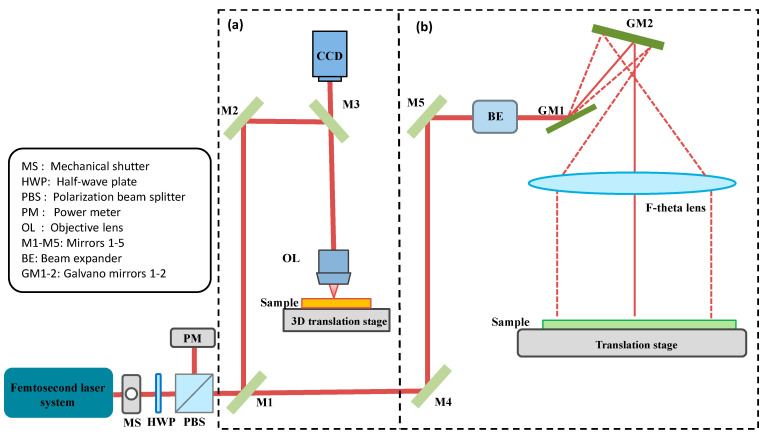
A typical setup of FsLDW system with (**a**) an objective lens and (**b**) a Galvo scanning system.

Laser direct writing offers great flexibility with non-contact and maskless fabrication processes, significantly reducing in manufacturing costs. By combining local processing with patterning in a single step, laser direct writing greatly enhances manufacturing efficiency. The production of two-dimensional (2D) and three-dimensional (3D) microstructures through femtosecond laser direct writing (FsLDW) is typically achieved in two ways: either by moving the 3D transformation stage or by using a galvanometer combined with the transformation stage, as depicted in Figure 2. The former method is suitable for small-scale, high-precision machining tasks. Nevertheless, it remains slower in accomplishing rapid and adaptable micromachining of complex microstructures on a large scale. Owning to highly developed high power, high repetition frequency, and miniaturized femtosecond laser, scanning galvanometers (galvo) are adopted to achieve high throughput and high-resolution micromachining, which is beneficial for the commercialization of femtosecond laser micromachining.

Most current femtosecond lasers use Ti: sapphire or Ytterbium fiber as the gain medium. Low energy and high repetition rate femtosecond pulses are first generated in a mode-locked oscillator. Typical commercial Ti: sapphire laser systems can produce pulses with tens of fs duration at about 800 nm wavelength and up to 250 kHz repetition rate [30]. The ytterbium fiber, on the other hand, can deliver power in the range of a few watts to a hundred watts with pulse widths of 0.2 to 10 ps and up to 100 MHz repetition rate [31,32]. These pulses are then amplified to microjoule and millijoule levels for machining applications using a technique known as chirped pulse amplification [33]. In the beam control section, a high-speed optoelectrical shutter is used to control the number of pulses, and the combination of a half-wave plate and polarizer is used to adjust the pulse energy. By using a high numerical aperture objective and a 3D translation stage, laser direct wiring has manufacturing resolution down to the sub-micron range. The FsLDW setup with galvo scanning system is used for fast and large-area processing. Finally, the FsLDW is controlled by a computer with the necessary application software to control various devices. Sample motion and beam scanning parameters must be controlled according to the machining requirements.

### 3.2. Spatial–Temporal Pulse Shaping Processing

**Figure 3:**
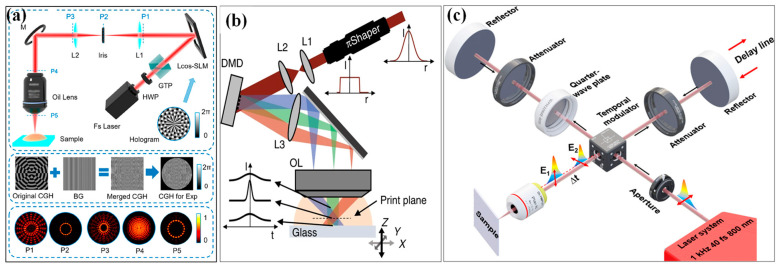
Illustrations of optical systems and principles for parallel processing systems based on (**a**) LC-SLM (adapted with permission from [45]. Copyright @ 2019 American Chemical Society) and (**b**) DMD (reprinted with permission from [39] on the basis of open access), (**c**) Schematic diagram of the experimental setup for generating the peculiar hybrid grating-hole nanostructures on the surface of Fe-based metal glass upon irradiation of the space–time modulation of femtosecond laser pulses (red double arrows represent the direction of the laser polarization; red single arrow for the delay-line movement; and black single arrow for the direction of the laser propagation). (Reprinted with permission from [56], Copyright © 2022, American Chemical Society).

To address the development of various nanomaterials and relevant devices, various optical modulation processing methods have been invented to increase the efficiency and flexibility of complex microstructure fabrication, including multifocal parallel and plane exposure processing by diffractive optical components (DOE) [34,35], liquid crystal spatial light modulator (LC-SLM) [36,37], and digital micromirror device (DMD) [38,39,40]. For most applications, LC-SLMs and DMDs are popular spatial shaping methods because of their ability to dynamically adjust the processing graphics. LC-SLM modulates the amplification/phase of the optical field by changing the distribution of liquid crystals. Satoshi et al. [41] first proposed the combination of LC-SLM and femtosecond laser processing. Combined with the theoretical approach of genetic algorithm, the computer-programmed control can achieve the expected desired shaping pulse of any shape, such as Bessel beam [42], vortex beam [43], etc. As a flexible micro-patterning method, femtosecond laser processing based on SLM has attracted much attention in recent years. The current SLM parallel machining methods are mainly multi-focus machining and face exposure machining. Hu et al. [44] used an SLM-based femtosecond laser multifocal parallel scanning technique, which utilized the eight generated parallel foci to process the micropillar at high efficiency. Wang et al. [45] designed a Matthew beam generated by the phase SLM to fabricate complex micro-cages, as shown in Figure 3a. This processing method has a relatively high processing resolution and ultra-high processing efficiency. In contrast to the aforementioned techniques, Yang et al. [46] developed a focal field engineering method that utilizes the Gerchberg–Saxton algorithm to generate axial cross sections of 3D structures. These cross sections are then machined to produce Fresnel microlenses with continuous surfaces, as well as other 3D structures. In addition, researchers have used SLM to change the phase to increase the depth of focus or to introduce multiple spots. In 2013, Hu’s group [47] introduced phase-only SLM into a conventional FsLDW system to actively extend the focal depth of a tightly focused spot along the beam propagation direction without significantly sacrificing numerical aperture. The on-axis intensity of deep-focus beams can exceed the processing threshold over a long enough range to obtain high-aspect-ratio voxels. This strategy was used for the precise and rapid fabrication of mesoscale binary optical elements with microscale characteristics. Recently, Naohiro et al. [48] demonstrated the treatment of self-suspended monolayer graphene with a spatial reshaping femtosecond laser using SLM, which enables multi-point drilling of holes with diameters smaller than 100 nm. The SLM is used as a four-fold symmetric 2D phase grating to realize multi-point processing with interference of multiple beams. Graphene atomic defects can be formed by such a processing technique, which will contribute to the future development of new graphene-based flexible devices. Accordingly, using SLM are beneficial in improving processing efficiency and facilitating patterning. However, they have several disadvantages. These include a narrow operating bandwidth and high cost. Additionally, the SLM suffers from low diffraction efficiency and limited integration capability. Additionally, the use of liquid crystal technology in spatial light modulators restricts the refresh frequency and makes it difficult to apply in high-power laser scenarios.

In addition, DMD, as another spatial light modulator, uses an array of micromirrors to control the reflection of light, which can modulate the light field into arbitrary two-dimensional patterns. In general, compared to LC-SLM, DMD can offer a higher refresh rate, lower cost, wider bandwidth, and a relatively high damage threshold. Due to its unique advantages, DMD-based femtosecond laser processing is characterized by high throughput, high contrast, fast response time, and ease of use. Wang et al. [38] proposed a protocol to optimize the consistent pattern printing of gap structure in femtosecond laser DMD projection lithography. They investigated the relationship between the structure morphology and the light intensity distribution at the image plane by multi-slit diffraction model and Abbe imaging principle. The continuously adjustable structural gap widths of 2144 nm, 2158 nm, and 1703 nm, corresponding to 6, 12, and 24 pixels, respectively, were obtained by varying the exposure energy. In 2019, Sourabh et al. [49] proposed a novel spatial–temporal synchronous focusing approach based on DMD for additive manufacturing. This method enables parallel processing of highly complex 3D structures with super-resolution and high throughput. This increases the throughput up to three orders of magnitude, and the axial resolution can be as high as 175 nm. Multi-photon lithography (MPL) with femtosecond laser is the dominant additive manufacturing technique for 3D printing at the micro/nanoscale. However, the low throughput of the typical point-by-point MPL process often limits its application. Therefore, there has been a focus on improving MPL’s printing rates. Paul et al. [39] present the development of a rapid and continuous projection MPL system with femtosecond laser, as shown in Figure 3b. The system utilizes a 5 kHz femtosecond laser beam that is spatially modulated in amplitude through a DMD to achieve high patterning rates of an entire image. The ultrafast laser pulses enable the fabrication of thin, solid layers by means of their spatiotemporal focusing and imaging effect. Smooth and continuous 3D objects can be printed rapidly by synchronizing the DMD patterns and axial stage motion. In 2023, Jungho et al. [40] used a similar projection-based patterned femtosecond with a DMD for a two-photon reduction technique, which can print arbitrarily complex 2D patterns at one time. Despite the high throughput, speed, and efficiency of DMD-based femtosecond laser processing, significant energy loss occurs due to the diffraction of the incident laser in the DMD. Consequently, the processing system requires high-power femtosecond laser light sources, yet energy utilization remains low.

Besides, temporal shaping turns a single laser pulse into a sequence of sub-pulses with a specified time interval and an arbitrary intensity ratio. To satisfy different processing requirements, temporal shaping can be used to adjust the pulse delay of the sub-pulses, the number of pulses and the energy ratio between the sub-pulses. Typically, the devices used to implement temporal shaping of a femtosecond laser are either commercial temporal pulse shapers based on a 4*f* system [50], or multi-pulse shapers based on a Michelson interferometer [51] and birefringent crystal [52], etc. It should be noted that the temporal and spatial shaping of the pulses can be performed independently or simultaneously. It is possible to control multiple pulse trains to create spatial interference in the light field, which, in turn, can be used to machine large areas of periodic structures. Femtosecond laser pulse interferometry has the advantages of high efficiency and a controllable period, which is an effective tool for constructing periodic functional micro-nanostructures [53,54]. For example, Li et al. [55] proposed a novel patterning method for mask-free and flexible fabrication of surface structures through a time-saving spatiotemporal-interference-based femtosecond laser shaping technique, based on a Michelson interferometer. By using this technique, fabrication of large-area surface structures and three types of terahertz filters are fabricated successfully. Zhao et al. [56] proposed a patterning method for the controllable formation of the grating-hole structures on metallic surfaces upon irradiation with spatiotemporally modulated femtosecond lasers, i.e., orthogonal linear polarizations of time-delayed double laser beams are manipulated into a tightly spatial energy distribution by optical diffraction, as shown in Figure 3c. The surface structure induced by the laser not only appears highly homogeneous, but also consists of arrays of nanopores with a uniform distribution of subwavelength gratings. This spatiotemporally modulated laser-induced structure offers a feasible method for manipulating optical response and detection in micro-optical components as needed. Furthermore, this temporal shaping approach, such as changing the delay time of a double pulse, can induce transient electronic excitation. This allows for the modulation of the physical–chemical properties of the flexible electrode precursor material [57,58].

### 3.3. Femtosecond Laser Sintering Technology

Laser sintering is a process where multiple nanoparticles are bonded together to form a network structure by the photothermal effect [59]. The pulse duration is one of the most important parameters affecting the nature of the laser–material interaction and its associated effects. Continuous wave and nanosecond lasers are widely used for sintering, but they cause undesirable thermal damage, large heat affected zones (HAZ), and low resolution. Femtosecond lasers have great potential because their pulse duration is much shorter than the duration of the electron lattice relaxation process. The highly localized energy distribution can significantly reduce HAZ. Femtosecond laser sintering is mainly achieved by the plasma resonance effect of metal nanoparticles excited by an intense laser. By controlling the process parameters of the femtosecond laser, the thermal field can be controlled to achieve precise sintering of nanoparticles.

**Figure 4:**
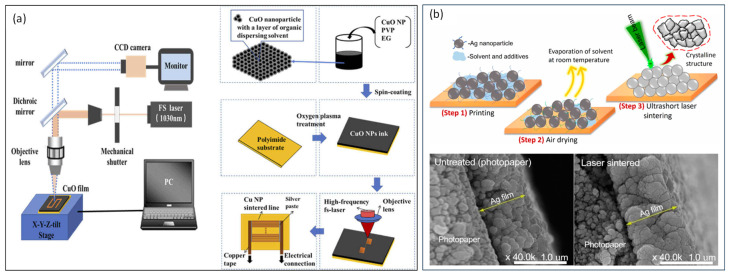
Electrical connection is created by femtosecond laser sintering of nanoparticles. (**a**) Femtosecond laser sintering copper nanoparticles (adapted from [63] on the basis of open access). (**b**) Schematic illustration of experimental strategy involving Ag NP printing; air drying at room temperature and femtosecond laser sintering steps; and the cross-section images of printed Ag NPs on photopaper, revealing the variations in film morphology before and after femtosecond laser sintering (adapted from [59] on the basis of open access).

Silver metal has high electrical conductivity and good chemical stability, making it a common electrode material for flexible optoelectronic devices. The presence of solvents and precursors in nanoparticles-based inks affects the electrical conductivity of the printed pattern. A post-processing heating step is typically performed to restore the electrical properties and structure of the material. Zhou et al. [60] effectively sintered silver nanoparticles onto silicon wafers using a low-fluence femtosecond laser. However, it was noted that at high optical fluences, the silver nanoparticles fused into larger particles when they melted due to femtosecond laser irradiation. Kim et al. [61] studied the impact of fluence on the sintering of silver nanoparticles. They found that low fluence helps connect neighboring silver nanoparticles, while higher fluence can cause melting or spheroidization, leading to a significant reduction in the mechanical strength of the electrodes. Copper is frequently used as an electrode material because of its high conductivity and affordable price. Peng et al. [62] utilized an 800 nm femtosecond laser to sinter copper nanoparticles, resulting in a minimum cubic resistance of 11.2 Ω/sq for the prepared copper electrode. Huang et al. [63] used a femtosecond laser with a frequency of 76 MHz to reductively sinter copper electrodes on polyimide films, and they obtained copper wires with a width of 5.5 μm and a porosity of 9.89% by optimizing the scanning speed, and the copper wires achieved a purity of 91.42% and a resistivity of about 1.3 × 10^−7^ Ω/m, as shown in Figure 4a. Mizoshiri et al. [64] sintered copper oxidation with a protection gas (nitrogen or argon), effectively inhibiting copper oxidation. In 2023, Sharif et al. [59] reported the printing of picoliter droplet volumes of Ag NPs onto flexible substrates using an acoustic microdroplet dispenser. Printed Ag patterns are sintered using a femtosecond pulse laser with minimal heat affected zone. After femtosecond laser sintering, the resistivity of Ag NP film reaches a value of 1.35 × 10^−7^ Ω m on photopaper. Meanwhile, no film/substrate damage was observed due to the near-negligible heat-affected zone of the femtosecond laser, as shown in Figure 4b. Therefore, the facile, selective, and controlled printing and femtosecond laser sintering of nanoparticles is cost-effective and provides better control to tune the structural and electrical properties of the sintered material, which are essential for the flexible electronic device applications.

### 3.4. Femtosecond Laser Reduction Technology

**Figure 5:**
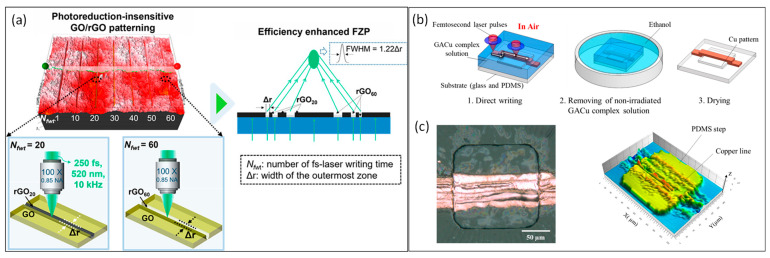
Femtosecond laser reduces GO and CuO nanoparticles to prepare electrode materials. (**a**) Illustration of photoreduction-insensitive GO/rGO patterning. (Reprinted with permission from [68], Copyright © 2021, American Chemical Society) (**b**,**c**). Schematic illustration of the femtosecond laser direct writing on glass or PDMS substrates, and the optical microscope image of the patterns on a PDMS step structure (top view) and 3D mapping of the fabricated Cu pattern (adapted from [74] on the basis of open access).

Femtosecond laser reduction is often employed in graphene oxide (GO) or metal oxides to prepare electrode materials or sensing materials for flexible devices-. As a kind of GO derivative, the laser reduction of GO has led to the development of graphene-based flexible electronics due to its high specific surface area and unique electro-optical properties. This is currently one of the best methods to prepare graphene in the laboratory. Femtosecond laser can achieve the reduction of GO to obtain reduced graphene oxide (rGO) [65], and the process involves photochemical reactions, photothermal reactions, and photoablation [66]. Zhang et al. [67] demonstrated that the resistivity and conductivity of reduced graphene are highly dependent on the output power of the femtosecond laser. They also successfully prepared graphene microcircuits by reducing GO with a femtosecond laser at a central wavelength of 790 nm and a pulse width of 120 fs, which laid the foundation for the application of graphene materials in flexible electronic devices. Lee’s group [68] proposed a multistep femtosecond laser writing approach to generate photoreduction-insensitive patterning of GO/rGO, as shown in Figure 5a. The number of femtosecond laser writing times can be used to control the degree of photoreduction of GO/rGO without altering the patterning linewidth, which only relies on pulse energy. The proposed method for patterning provides valuable insights into interactions between femtosecond lasers and GO, as well as effective solutions to the trade-off between performance and miniaturization in devices based on GO and rGO. Furthermore, femtosecond lasers can reduce metal oxide nanomaterials or ionic metal salt precursors to metal nanoparticles at low pulse energies [69,70]. Compared with other metal materials, copper has good electrical and thermal conductivity, while its abundant natural reserves and low cost have made it one of the widely used metal materials [71,72]. Taking copper-based oxides as an example, femtosecond lasers can reduce CuO nanoparticles to Cu NPs with the assistance of reducing agents (ethylene glycol [63], or polyvinylpyrrolidone [73]), and finally, Cu NPs are remelted into Cu circuits by laser sintering, and the whole process is mainly the result of photothermal and photochemical effects. Nam et al. [74] successfully prepared copper patterns on polydimethylsiloxane (PDMS) substrates using femtosecond laser-induced reduction of glyoxylic acid copper complex, as shown in Figure 5b,c. The minimum resistivity of the patterns on PDMS substrates is 1.4 × 10^−5^ Ωm, which is 10 times higher than that on glass substrates, indicating that this technology is useful for fabricating flexible microdevices. In addition, the femtosecond laser can also enable the reduction of ionic gold, silver, and polymetallic ions such as Ag^+^/Pd^2+^ to obtain Au and Ag NPs [75] and cobalt oxide nanoparticles [76]. Femtosecond laser reduction of metallic or nonmetallic oxide materials is more economical because metallic or nonmetallic oxide materials are more readily available than single-element metallic materials, are more stable in atmospheric environments, and have an advantage preparing future large-scale flexible circuits. However, femtosecond laser reduction of metallic or non-metallic oxides may have insufficient reduction or re-oxidation after reduction, resulting in poor performance of the conductive circuits prepared by femtosecond laser reduction, and there is still a need to explore the process of femtosecond laser reduction of metallic or non-metallic oxide inks to obtain conductive circuits with excellent electrical and mechanical properties.

### 3.5. Femtosecond Laser Nanojoining

Recent advances in ultrafast pulsed laser technology, particularly in femtosecond laser welding, have marked a paradigm shift in manufacturing science. Femtosecond laser welding leverages extremely brief pulses to achieve precise, localized energy deposition in materials, which is critical for preserving the integrity of sensitive nanomaterials [77,78]. The distinguishing feature of these pulses is their highly localized effects, which negate thermal damage and maintain the unique properties of nanoscale materials [23,27].

**Figure 6:**
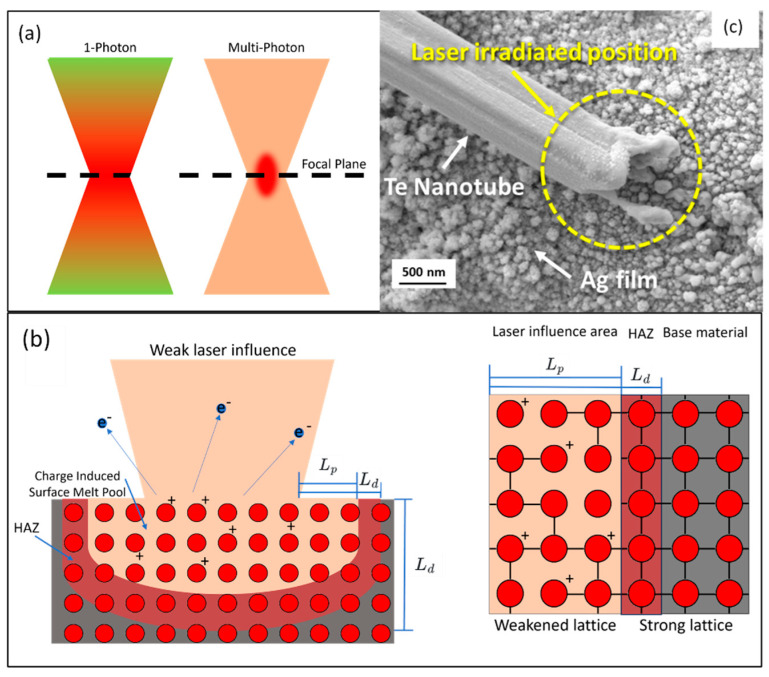
(**a**) Comparative illustration of single-photon and multi-photon absorption in femtosecond laser processes. (**b**) Demonstration of femtosecond influence on surface melting features. (**c**) SEM images of randomly distributed tellurium nanotube on printed silver film electrodes after femtosecond laser irradiation with 25 mW (adapted from [79] on the basis of open access).

Femtosecond laser welding operates through advanced non-thermal mechanisms, prominently including multiphoton absorption, ionization, and surface melting, which is depicted in Figure 6. Ultrashort multi-photon absorption first enables the material to absorb energy from intense femtosecond laser pulses without significant heat generation. As mentioned in Section 2, the multi-photon effect is confined at the vicinity of the focal point (~the diffraction limit, Figure 6a). The absorbed energy ejects electrons from surface atoms, with a skin thickness equal to the light penetration depth $L_{p}=1/\alpha$, where the optical absorption factor α can be determined by the *Beer–Lambert* equation, $I=I_{o}e^{-\alpha z}$, where *z* is the light path. At an extremely high laser influence, violent electron ejections cause the Coulomb explosion of large amounts of positive charged ions; however, at a low laser influence, low amounts of electron vaporization may only decrease the bond strength of surface atoms. This decreasing of atomic bonding may cause the surface melting with a skin depth of *Lp* (Figure 6b) [26].

As electrons are ejected from the material’s surface, electron density will decrease near the atoms, which, in turn, weakens the interatomic bonds [23]. This weakening of atomic bonds does not necessarily lead to immediate material removal but can result in a phase transition, i.e., atoms remarkably increase the mobility, like in a liquid. Unlike traditional thermal melting, this quasi-liquid state is not caused by a rise in the temperature of the lattice (the vibration of the ordered structure of the atoms), but by changes in the electronic structure of the material. The material in the affected region becomes less ordered, transitioning to a liquid-like state while the bulk of the material remains solid and largely unaffected. Concurrently, the coupling thermal from electrons to lattices will diffuse into the material. This thermal diffusion length is characterized by $L_{d}=\sqrt{D\tau_{p}}$, where *D* is the heat diffusion coefficient, and *τ_p_* is the laser pulse width [27]. This *L_d_* determined HAZ (Figure 6b). According to the experiment, this HAZ is only a couple of µm [28,30]. This localized surface melting and HAZ are advantageous in nanojoining, where highly localized thermal effects minizine the influence of the geometry and properties of nanomaterials, as shown in Figure 6c. Nanojoining facilitates subtle modifications at the molecular or atomic levels, which is essential for delicate alterations in nanomaterials [8,14]. The operational efficacy of this welding technology hinges on various parameters: pulse duration, laser energy, focus, spot size, repetition rate, and wavelength [80,81,82]. Tailoring these parameters is crucial for achieving precise energy deposition at nanoscale regions and for optimizing energy transfer, especially in applications demanding high spatial resolution with minimal collateral effects.

Nanojoining enables the precise joining of materials such as nanowires, nanoparticles, and thin films, which is instrumental in the development of a wide array of nano-devices and structures. This technology stands out in its ability to connect nanowires and fabricate nanoscale electronic circuits, a task where traditional welding techniques falter due to their inability to preserve the structural integrity of ultrafine structures. Additionally, in the context of nanoparticle fusion, femtosecond lasers excel at merging nanoparticles to form larger aggregates or attaching them to diverse substrates, a capability that is increasingly vital in the advancement of sensor technology [80,83]. In addition, femtosecond laser irradiation has proven to be a transformative tool for enhancing nanojoining in memristor and nanoelectronics devices [79,84].

### 3.6. Femtosecond Laser-Induced Graphitization and Carbonization

**Figure 7:**
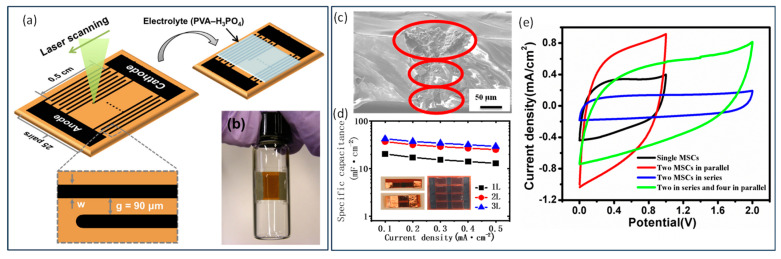
(**a**) Schematic of the micro-supercapacitor fabricated by laser carbonization. (**b**) Photographic image of a fabricated micro-supercapacitor attached to the curved wall of a vial (r = 7.5 mm). (Reprinted from [87], Copyright © 2014 Elsevier Ltd.) (**c**) Cross-section of the three-layer electrode. The circle stands for the carbonized area. (**d**) Specific capacitances of the MSCs calculated from the GCD curves as a function of the current density. Photos of the fabricated MSCs with different connections are inserted at the bottom. (**e**) GCD curves of MSCs with four kinds of connections at a current density of 0.1 mA/cm^2^. (Adapted from [90], Copyright © 2017 Elsevier Ltd.).

The femtosecond laser can produce instantaneous high temperature and high pressure due to its extremely high power density, which can graphitize or porously carbonize some polymer materials (such as polyfluorene, polyimide, lignin, cellulose, wood, etc.) directly and assist in the preparation of flexible circuits and electronic devices [70,85,86,87]. The mechanism of femtosecond laser-induced graphitization or porous carbonization of polymeric materials is more complex, and the detailed mechanism is not yet known, but it is still a photothermal or photochemical process, or both [88]. Morita et al. [89] reported the discovery of a localized photoconversion to a graphite-like structure inside polyfluorene derivatives formed of 3D cardo structures generated by powerful ultrashort light pulses. Such space-selective photoinduced microstructures exhibit high electrical conductivity of about 30 S/m. In addition, polyimide (PI) films, due to their flexibility, excellent mechanical properties, high-temperature resistance, and corrosion resistance, are currently the ideal flexible substrate material for manufacturing flexible electronic devices. The femtosecond laser focusing position can induce multiphoton absorption to make PI local temperature up to 1000 K or more, leading to melting and boiling, decomposition, and carbonization to generate porous carbon structure [90]. Bin In et al. [87] prepared a very flexible all-solid-state micro-supercapacitor (MSC) using this process (Figure 7a), and systematically investigated the effects of laser power, scanning speed, and the number of scans on the conductivity of porous carbon structures. The prepared flexible MSC has a specific capacitance of about 800 μF/cm^2^ at a test rate of 10 mV/s. Wang et al. [90] induced the generation of multilayer by regulating the distance between the objective and the polyimide film to achieve the fabrication of 3D MSC with an area-specific capacitance of 42.6 mF/cm^2^ at a current density of 0.1 mA/cm^2^, as shown in Figure 7c–e. In addition, Wang et al. [91] reported transforming lignin into porous conductive carbon structures and interdigitated circuits for supercapacitor devices using FsLDW. Morosawa et al. [88] demonstrated the fabrication of highly conductive graphitic carbon from cellulose nanofiber films using a high-repetition femtosecond laser. By scanning the laser beam once, a conductivity as high as 6.9 S/cm was attained, which is believed to be more than 100 times greater than the previously recorded conductivity. They hypothesize that, in the case of highly repetitive femtosecond laser irradiation, strong optical effects, in addition to thermal effects, contribute to the degradation of CNFs, resulting in the formation of highly crystalline graphitic carbon, which contributes to the formation of high electrical conductivity. These works demonstrate that femtosecond laser irradiation of polymeric materials, such as PI, leads to the formation of carbon structures doped with nitrogen and oxygen atoms, which enhances the pseudocapacitance of supercapacitors compared to continuous lasers [90]. Furthermore, the surface porous carbon-based structures induced by femtosecond laser also demonstrate piezoresistive properties and negative temperature characteristics similar to semiconductors, making them suitable for use as pressure and temperature sensors [92]. Additionally, the use of femtosecond laser for electrode induction results in smaller feature sizes, which is more conducive to device integration [93].

## 4. Applications in Flexible Electronic Devices

The development of flexible electronic devices is extensively focused on diversification, integration, and multi-functionality for portable and wearable electronics. Various flexible electronic devices are invented and listed as follows: flexible energy storage devices, flexible triboelectric nanogenerators, flexible sensors, flexible detectors, and other flexible devices (phase modulation, super lens, light absorption device, etc.). As some of the applications are still in the primary research stage, there are still many process optimizations to be explored, so we will briefly discuss in the progresses in these fields.

### 4.1. Flexible Energy Storage Devices

**Figure 8:**
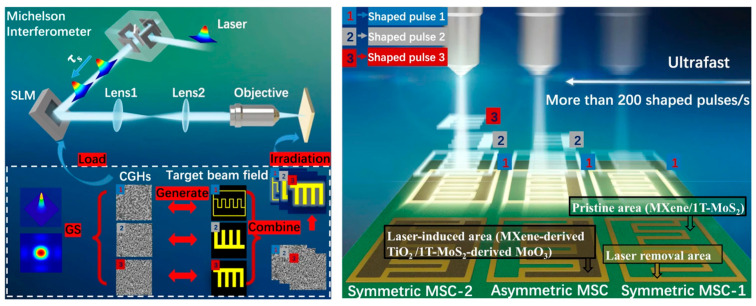
Schematic diagram of the SLM-based maskless patterning method for ultrafast manufacturing of multitype MSC (reprinted from [58] on the basis of open access).

Supercapacitors are important members of electrochemical energy storage devices, which have received wide attention by their high-power density, ultra-long cycle life, and other advantages [94,95,96]. At present, the electrode preparation in conventional supercapacitors still uses the slurry coating technique [97,98]. However, this process technology is highly dependent on the composition of the slurry, the formulation conditions, and the collector fluid, resulting in inefficient mixing of electrode materials, as well as difficult control of the interfacial composition, which makes it difficult to efficiently exploit the electrochemical properties of the active materials. With the development of nanotechnology, electrostatic spinning, photolithography, 3D printing, laser direct writing, and other industrialized technical means of electrode preparation are considered the most promising technical means in the future [99,100,101,102,103,104]. Among them, femtosecond laser-based direct writing technology has been a hot research topic [105,106,107,108]. Li et al. [105] prepared patterned rGO and gold collector structures for micro-supercapacitors using laser direct writing of in situ reduced GO and chloroauric acid (HAuCl_4_) nanocomposites. FsLDW simultaneously reduces GO and chloroauric acid to rGO and gold nanoparticle collectors. The nano-connected gold nanoparticles significantly increase the specific surface capacitance of porous graphene while increasing its electrical conductivity. Due to the gold nanoparticles, the conductivity increases to 1.1 × 10^6^ S/m and its specific surface capacitance can reach 4.92 mF/cm^−2^ at 1 V/s scanning speed. Yuan et al. [57]. demonstrated a dual femtosecond pulsed laser carbonization-deposition method for preparing carbon-based electrodes and successfully deposited porous amorphous carbon, graphene, and carbon quantum dots with controlled properties by temporally controlling the femtosecond laser. The resulting MSC had a very high-frequency response and performed well at scan speeds up to 10,000 V/s. The MSC’s characteristic frequency *f*_0_ reached 42,000 Hz, while the relaxation time constant *τ*_0_ was 0.0238 ms. At a frequency of 120 Hz, the MSC achieved an impedance phase angle of −82.6 degrees, an ultrahigh power density of more than 30 kW cm^−3^, and an energy density of 0.068 W h cm^−3^. This technology opens a new avenue for the development of ultrahigh frequency filters for future tiny portable electronic devices. However, the above-reported MSCs prepared by FsLDW were mainly produced from non-degradable synthetic polymers, which may result in electronic waste. Recently, Young-Jin Kim et al. [106] reported the direct fabrication of highly conductive, intrinsically flexible, and green microelectrodes from naturally fallen leaves in ambient air using femtosecond laser pulses. The sheet resistance of the microelectrodes generated on leaves is lower (23.3 Ω/sq) than that of their synthetic polymer counterparts, and the MSCs have an excellent areal capacitance (34.68 mF cm^−2^ at 5 mV/s) and capacitance retention (about 99 percent after 50,000 charge/discharge cycles). The FsLDW MSCs on a single leaf have the potential to be used in wearable electronics, smart homes, and as part of the Internet of Things. In 2023, Jiang’s group [58] developed a maskless ultrafast fabrication of multitype micron-sized (10 × 10 μm^2^) MSCs via temporally and spatially shaped femtosecond laser, as shown in Figure 8. The original Gaussian laser is converted into a double pulse with pulse delay by the Michelson interferometer. It then passes through the SLM and is transported to the objective lens by the 4*f* system to realize micro/nano processing. The magnified image of the objective and the sample can be processed in an extremely short time by controlling the 1, 2, and 3 subpulses to obtain various types of MSCs. MXene/1T-MoS_2_ can be integrated with laser-induced MXene-derived TiO_2_ and 1T-MoS_2_-derived MoO_3_ to generate over 6000 symmetric MSCs or 3000 asymmetric micro-supercapacitors with high-resolution (200 nm) per minute. The asymmetric micro-supercapacitors can be integrated with other micro devices, thanks to the ultrahigh specific capacitance (220 mF cm^−2^ and 1101 F cm^−3^), voltage windows in series (52 V), energy density (0.495 Wh cm^−3^), and power density (28 kWcm^−3^). This approach enables the industrial manufacturing of multitype micro-supercapacitors and improves the feasibility and flexibility of micro-supercapacitors in practical applications. From these works, it can be seen that femtosecond laser processing of energy storage devices does not only favor the study of inducing different material systems, such as PI film or lignin, but also more precisely modulate the physical properties of the electrode materials, like realizing highly conductive electrodes, high specific surface area electrodes, combinations of different functional materials and highly refined electrode structures, to further enhance the properties of flexible energy storage devices.

### 4.2. Flexible Triboelectric Nanogenerator

**Figure 9:**
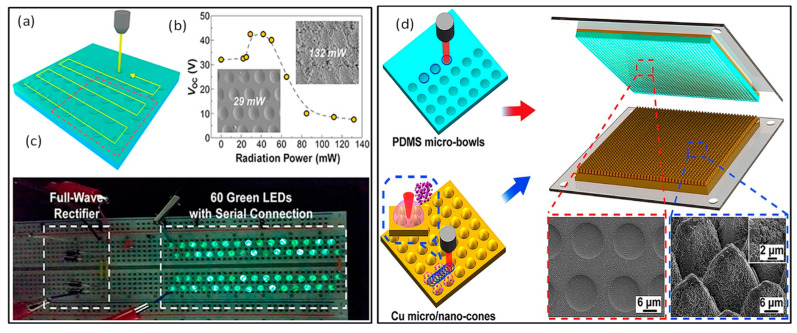
(**a**) Schematic illustration of the fabrication of the PDMS by femtosecond laser irradiation; (**b**) open-circuit voltage of the fabricated TENGs with laser power ranging from 0 to 132 mW; (**c**) a digital camera snapshot showing 60 serially connected green LEDs lit simultaneously. (Reprinted from [111], Copyright © 2017 Elsevier Ltd.); (**d**) schematic illustration of TENG based on femtosecond laser ablation of Cu micro/nano cone structure and PDMS micro bowl structure. (Reprinted from [112], Copyright © 2019 Elsevier Ltd.).

The triboelectric nanogenerator (TENG), is a novel energy harvesting technology based on interface contact electrification and electrostatic induction [109,110]. Improving the surface roughness and contact area of the friction layer helps to generate more frictional charges, as well as increase capacitance and effective dielectric constant during contact, which is one of the most effective ways to improve TENG performance and can be accomplished through femtosecond laser micro-nano-weaving. Kim et al. [111] used a femtosecond laser to induce micro-nano weaving on the surface of PDMS and used it as a friction layer for TENG devices, as well as investigating the effect of micro-nano structures on TENG performance at different laser powers (Figure 9a). The TENG using the PDMS patterned with laser power of 29 mW produced a maximum voltage of 42.5 V and a maximum current of 10.1 μA, along with a power density level of 107.3 μW/cm^2^. The constructed TENG shows exceptional durability and a high potential for usage as an electrical energy provider. Huang et al. [112] used the FsLDW technique to prepare cone-like composite micro/nanostructures by ablating on the surface of copper thin film and obtained micro bowl-like structures by ablating on the surface of PDMS, and used the above two structures as friction layers to construct TENG, and the preparation flow is shown in Figure 9b. The TENG with micro/nanostructure achieves instantaneous power of 13.99 μW at 10 MΩ and 21 times enhancement in power density than that of the TENG without micro/nano-structures. Recently, Zhang et al. [113] proposed a droplet triboelectric nanogenerator with a superhydrophobic surface and self-cleaning capability fabricated by femtosecond laser direct writing. The droplet TENG with laser treated polytetrafluoroethylene (PTFE) dielectric layer can reach an open-circuit voltage of 60 V and a short-circuit current 3.2 μA after a full pre-charge, which are 3 and 1.5 times improved compared with those of the TENG with a PTFE dielectric layer, respectively. The droplet TENG also demonstrated good long-term stability, self-cleaning ability, and flexibility, making it suitable for various applications. Therefore, the design and preparation of nanogenerators provides a new strategy for improving the friction characteristics of dielectric material surfaces. Additionally, multifunctionalizing and arraying them by laser direct writing allows for their application in various complex environments. This lays a solid foundation for large-scale applications of TENG.

### 4.3. Flexible Wearable Sensors

**Figure 10:**
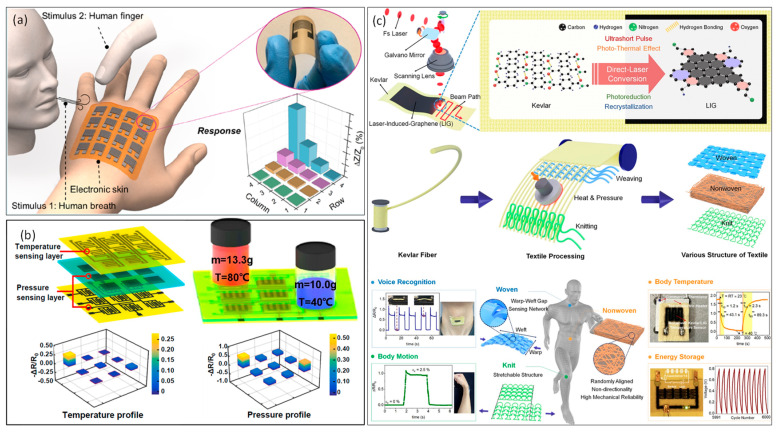
(**a**) Conceptual image of a flexible sensing matrix that conformably contacts with a human hand and provides viable responses to moisture stimuli, and the relative change in impedance (ΔZ/Z_0_) of each pixel in the matrix, demonstrating the mapping capability of the e-skin in the non-contact mode. (Reprinted from [118], Copyright © 2017, American Chemical Society). (**b**) A schematic of the sensor matrix prepared by the femtosecond laser-based micro-fabrication method, and the schematic illustrating the simultaneous mechanical and thermal measurements of the sensor matrix. (Reprinted from [92], Copyright © 2019 Elsevier Ltd.) (**c**) Schematic illustration of e-textile production enabled by one-step maskless patterning of LIG on woven, nonwoven, and knit textiles. Reprinted from [121], Copyright © 2023, American Chemical Society.

With the ongoing advancement of flexible electronics and blooming nanomaterials preparation technologies, innovative flexible strain sensors based on diverse functional nanomaterials and flexible substrates have attracted the attention of researchers [114,115,116,117]. Laser processing technology enables the fabrication of tiny, integrated high-performance flexible strain sensors. Laser peeling, laser transfer, and laser direct writing methods have been widely employed in the field of smart wearables in recent years. Due to their “cold processing” features, femtosecond lasers can deposit and shape diverse functional materials with adjustable feature sizes directly on flexible substrates. An et al. [118] used FsLDW of rGO patterns as electrodes and fabricated all-graphene-based highly flexible non-contact electronic skins, which exhibited high sensitivity, fast response-recovery behavior, and good long-term stability, as shown in Figure 10a. By taking the merits of the FsLDW, a 4 × 4 sensing matrix was facilely integrated with a single-step and showed high-spatial-resolution sensing capabilities over a long detection range in a non-contact mode. A flexible sensor matrix with functionalities for multiple stimuli detection has prospect applications in structural health monitoring, the Internet of Things, soft robotics, etc. To address this, Bai et al. [92] developed a femtosecond laser microfabrication method to fabricate a sensor matrix integrated with temperature and pressure sensor arrays. The temperature sensors have a temperature coefficient of resistance of 0.52%/°C, with a response speed of ~8 s. The pressure sensors delivered a sensitivity of −2.01 kPa^−1^, a detection window from 0.001 to 80 kPa, a response speed of 0.030 s, and high mechanical stability, as shown in Figure 10b. Furthermore, related work has described the production of metallic electrodes such as silver and copper on flexible substrates with good conductivity and stability employing FsLDW-assisted manufacturing methods, and demonstrated their applicability in flexible heating, sensing, and motion monitoring [119,120]. In 2023, Young-Jin Kim et al. [121] reported the direct laser writing of e-textiles by converting raw Kevlar textiles to electrically conductive LIG via FsLDW in ambient air (Figure 10c). Wearable multimodal e-textile sensors and supercapacitors are used on different types of Kevlar textiles, including nonwoven, knit, and woven structures, by considering their structural textile characteristics. This direct laser synthesis of arbitrarily patterned LIGs from various textile structures could result in the facile realization of wearable electronic sensors and energy storage. Recently, Yi et al. [122] proposed an active pressure sensor with triboelectric nanogenerator on PDMS surfaces. They fabricated microcolumn arrays with gradient height on PDMS surfaces using femtosecond laser spatial–temporal shaping technology to shape Gaussian beam into double-pulse Bessel beams and supplementing it with wet etching and imprinting technology. The sensor with 2 µm microcolumns can maintain a high linearity (R2 ≈ 0.99996) with a sensitivity of 0.304 V·kPa^−1^, even when the detection pressure range reaches 0–330 kPa. These results highlight the potential applications of self-powered triboelectric pressure sensors for health monitoring and human machine interaction. Furthermore, an increasing number of studies have focused on the application of press/stress sensors [123,124], gas sensors [125,126,127], temperature sensors [128,129], etc. These studies focus on utilizing femtosecond laser processing to enhance the performance of a single sensor in terms of sensing sensitivity, application range, and long-term stability. However, there is a lack of balanced design between low power consumption and high performance, transmission and analysis of multimodal sensing signals, and human skin contact and usage safety (for wearable scenarios). These issues involve multidisciplinary cross-cutting challenges for future researchers, as well as opportunities for development.

### 4.4. Flexible Optoelectronics

**Figure 11:**
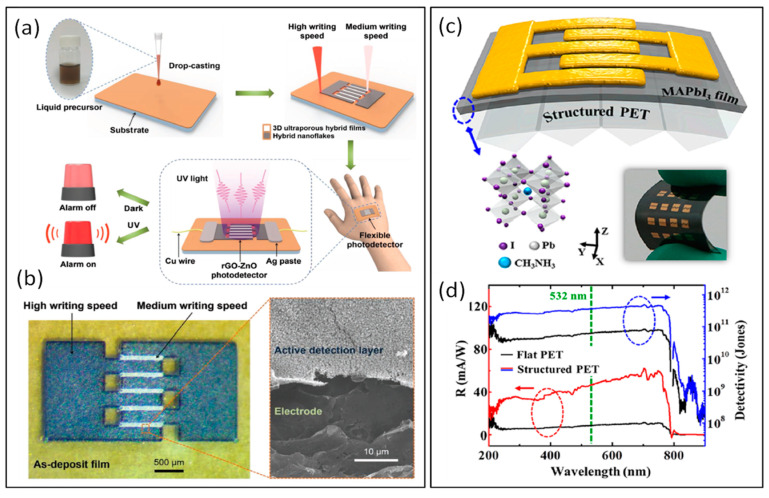
(**a**) Schematic illustration of the preparation of all rGO–ZnO hybrid-based photodetectors through a single-step FsLDW process by selecting a suitable writing speed for respectively patterning the interdigitated electrodes and the active detection layer. (**b**) In an optical image of the as-written interdigitated photodetector, an enlarged SEM image indicates the active detection layer and the electrode (Reprinted with permission from [132] on the basis of open access). (**c**) Schematic device structure of the perovskite FPD. The top-down materials are gold electrodes, MAPbI_3_ film, and structured PET substrate. The insets are the 3D schematic representation of MAPbI_3_ (left) and the optical image of the flexible device (right). (**d**) Comparison of photoresponsivity (R) and detectivity in the UV−NIR region. (Reprinted with permission from [133], Copyright © 2020, American Chemical Society).

Due to their exceptional photoelectric and mechanical qualities, flexible optoelectronics have drawn a lot of interest in recent years [130,131]. These characteristics can meet the demands of next-generation integrated devices for compatibility, functionality, and easy and affordable manufacturing. In 2018, Young-Jin Kim et al. [132] reported the synthesis of hierarchical hybrid nanocomposites, e.g., reduced graphene oxide (rGO)–zinc oxide (ZnO), using a femtosecond laser direct writing technique to finely tune the material properties by controlling the incident photon density, as well as building a highly flexible and all rGO–ZnO hybrid-based photodetector. The as-fabricated photodetector exhibited high, linear, and reproducible ultraviolet (UV) responsivities over a wide intensity range (0.6–20 mW/cm^2^) at a low operation voltage (1 V). The laser direct writing mechanism, the physical diagram of the hybrid composite, and the micro-nano device are shown in Figure 11a,b. To improve the photo-response of light-active materials, Wang et al. [133] developed a technique for achieving nearfield optical enhancement using periodic micron-sized grating structures made by femtosecond laser direct writing on the surface of poly (ethylene terephthalate). A CH_3_NH_3_PbI_3_ perovskite film and PET are selected as the light-active and base materials, respectively. Under a 1 V bias voltage actuation and 532 nm laser irradiation at an intensity of 10 mW/cm^2^, the flexible device exhibits excellent performance in photoresponsivity (47.1 mA/W), detectivity (3.7 × 10^11^ Jones), and on/off ratio (4600), as shown in Figure 11c,d. Flexible UV photodetectors (PDs) are in high demand for their wide range of applications in wearable devices. However, their complex fabrication techniques have limited their use. Recently, Liang et al. [134] utilized a FsLDW strategy to assemble SiC microwires within 30 s. The resulting 3D porous structure improves device responsivity. The microwire photodetector based on silicon carbide exhibited a responsivity of 55.89 A W^−1^ to 365 nm UV light at a bias of 1 V. Furthermore, the SiC microwires photodetectors was deposited directly on a flexible substrate and were able to operate stably even after 2000 bending cycles. This study reveals a feasible method to fabricate flexible circuits with excellent thermal stability and mechanical flexibility using FsLDW.

In addition, graphene, as a two-dimensional semi-metallic material with ultra-high carrier mobility, has a wide range of applications in the field of flexible optoelectronics. Structured graphene material surfaces created through femtosecond laser processing technology can enable functions that are beyond the inherent capabilities of the material itself. These functions include the manipulation of polarization and phase of light, and they contribute to advancing the practical application of graphene and other two-dimensional materials in flexible optoelectronic devices. Jia et al. [135] proposed a grating structure based on a femtosecond laser direct-written graphene and dielectric composite film, which was found to achieve light absorption in the broad spectral range of 300–2500 nm at an incidence angle of 0–60°. In addition, Yang et al. [136,137] successfully produced uniform subwavelength grating structures and simultaneous in situ photoreduction processes at high speed by exploiting the cylindrical focusing of a femtosecond laser on GO films. Further experiments demonstrated that such regular structured surfaces could enhance the light absorption (>20%) and birefringent response (~ 0.18 ratio) of the rGO films.

Furthermore, in flexible solar cells, transparent conducting oxides (TCOs) play a dual role of extracting photogenerated carriers and allowing sunlight to reach the photoactive material. Thus, it is crucial to control the electrical and optical properties of TCOs to optimize solar cell efficiency. Recently, Heffner et al. [138] employed a femtosecond laser interference patterning method to induce periodic linear microstructures on the surface of fluorine-doped tin oxide. The resulting microstructures have a period of 3.0 μm and an average height between 20 and 185 nm. As a result, the average total and diffuse light transmittance in the spectral range of 400–800 nm increased by 5% and 500%, respectively. The study confirms that femtosecond laser interference patterning method is a convenient technique for constructing electrodes for high-efficiency flexible photovoltaic devices.

## 5. Summary and Outlook

Although a number of inkjet printing, layer-by-layer assembly, screen printing, and conventional lithographic techniques have been developed for the preparation of flexible electronics, FsLDW becomes an innovative, scalable, contactless, and maskless 3D processing technology that enables high precision and high-quality processing preparation of flexible electrode materials. In addition, the combination of different processing methods, such as the parallel processing technique of spatial–temporal rectification, enables the preparation of patterned microstructures with high efficiency and high precision. Even in combination with the material design of precursors and the selection of processing atmosphere environment, the controllable fine doping modulation of heterogeneous atoms or elements can be realized, which, in turn, can enhance the performance of flexible electronic devices. This paper reviews the principle and characteristics of femtosecond laser processing, typical methods of femtosecond laser processing, and the status of its application for flexible electronic devices. As a non-contact technology, femtosecond laser micro and nano processing technology have comprehensive advantages of high processing accuracy, controllability, and high efficiency and integration due to the high peak power and small thermal effect of the femtosecond laser, which has great potential for application in the preparation of flexible electronic devices. In addition, the interaction between the femtosecond laser and the material allows fine-tuning of the electronic and optical properties of the material, providing a variety of possibilities for the development of multifunctional, highly integrated, and high-performance flexible electronic devices.

Flexible electronic devices fabricated using femtosecond laser micro and nano processing have been very promising for a variety of applications, but there are still several technological obstacles to overcome.

(1) The interaction mechanism between the femtosecond laser and materials is not yet completely clear. The specific process and mechanism of femtosecond laser-induced material reduction and carbonization still need to be studied in depth, such as the influence of nonlinear optical effects on the reduction and carbonization process.
(2) Several variables can affect the microstructures created by femtosecond lasers, making it challenging to control the chemical composition and optoelectronic characteristics of electrode materials accurately. The homogeneity of microstructures and rapid large-scale preparation are still challenging.
(3) For the preparation of flexible optoelectronic devices, femtosecond laser processing, although the rapid one-step preparation of flexible devices, the resulting material composition, structure, and precise regulation is more difficult so that the further optimization of device performance is hindered by the enhancement. In addition, multi-functional integrated flexible devices are an important future development direction. It is necessary to combine femtosecond laser direct writing technology or junction with electrochemical deposition, printing, lithography, and other auxiliary means to achieve the integrated design and manufacture of flexible electronic devices in energy storage, triboelectric nanogenerator, wearable sensors, photoelectric detection, and optical manipulation.

Although there are many challenges, femtosecond laser processing for flexible electronics has displayed great potential for application. This mini review may stimulate strategic methods to address technical issues by deepening the understanding of femtosecond laser–materials interaction.

## References

[1] Lin Z., Hong M. (2021). Femtosecond laser precision engineering: From micron, submicron, to nanoscale. Ultrafast Sci..

[2] Corrielli G., Crespi A., Osellame R. (2021). Femtosecond laser micromachining for integrated quantum photonics. Nanophotonics.

[3] Jia Y., Chen F. (2023). Recent progress on femtosecond laser micro-/nano-fabrication of functional photonic structures in dielectric crystals: A brief review and perspective. APL Photonics.

[4] Jianing L., Dongshi Z., Zhuguo L. (2022). Advance in femtosecond laser fabrication of flexible electronics. Opto-Electron. Eng..

[5] Bin D., Juan Z., Dawei W., Yiyuan Z., Leran Z., Rui L., Chen X., Shunli L., Zihang Z., Hao W. (2023). Femtosecond laser micromachining optical devices. Opto-Electron. Eng..

[6] You R., Liu Y., Hao Y., Han D., Zhang Y., You Z. (2010). Laser Fabrication of Graphene-Based Flexible Electronics. Adv. Mater..

[7] Gattass R.R., Mazur E. (2008). Femtosecond laser micromachining in transparent materials. Nat. Photonics.

[8] Lei S., Zhao X., Yu X., Hu A., Vukelic S., Jun M.B.G., Joe H.-E., Yao Y.L., Shin Y.C. (2020). Ultrafast laser applications in manufacturing processes: A state-of-the-art review. J. Manuf. Sci. Eng..

[9] Sundaram S.K., Mazur E. (2002). Inducing and probing non-thermal transitions in semiconductors using femtosecond laser pulses. Nat. Mater..

[10] Rethfeld B., Kaiser A., Vicanek M., Simon G. (2002). Ultrafast dynamics of nonequilibrium electrons in metals under femtosecond laser irradiation. Phys. Rev. B.

[11] Sun C.-K., Vallée F., Acioli L.H., Ippen E.P., Fujimoto J.G. (1994). Femtosecond-tunable measurement of electron thermalization in gold. Phys. Rev. B.

[12] Jiang L., Tsai H. (2005). Energy transport and material removal in wide bandgap materials by a femtosecond laser pulse. Int. J. Heat Mass Transf..

[13] Rethfeld B., Sokolowski-Tinten K., von der Linde D., Anisimov S.I. (2002). Ultrafast thermal melting of laser-excited solids by homogeneous nucleation. Phys. Rev. B.

[14] Shugaev M.V., Wu C., Armbruster O., Naghilou A., Brouwer N., Ivanov D.S., Derrien T.J.-Y., Bulgakova N.M., Kautek W., Rethfeld B. (2016). Fundamentals of ultrafast laser–material interaction. MRS Bull..

[15] Cheng C., Xu X. (2005). Mechanisms of decomposition of metal during femtosecond laser ablation. Phys. Rev. B.

[16] Cheng J., Liu C.-S., Shang S., Liu D., Perrie W., Dearden G., Watkins K. (2013). A review of ultrafast laser materials micromachining. Opt. Laser Technol..

[17] Petrakakis E., Tsibidis G.D., Stratakis E. (2019). Modelling of the ultrafast dynamics and surface plasmon properties of silicon upon irradiation with mid-IR femtosecond laser pulses. Phys. Rev. B.

[18] Sun M., Eppelt U., Schulz W., Zhu J. (2013). Role of thermal ionization in internal modification of bulk borosilicate glass with picosecond laser pulses at high repetition rates. Opt. Mater. Express.

[19] Bashir S., Rafique M.S., Husinsky W. (2012). Identification of ultra-fast electronic and thermal processes during femtosecond laser ablation of Si. Appl. Phys. A.

[20] Zhao X., Shin Y.C. (2013). Coulomb explosion and early plasma generation during femtosecond laser ablation of silicon at high laser fluence. J. Phys. D Appl. Phys..

[21] Bulgakova N.M., Zhukov V.P., Marine W., Vorobyev A.Y., Guo C. (2008). Charging and plasma effects under ultrashort pulsed laser ablation. High-Power Laser Ablation VII.

[22] Yang M., Li B., Deng G. (2022). Comparison study of the femtosecond laser-induced surface structures on silicon at an elevated temperature. Opt. Express.

[23] Stampfli P., Bennemann K.H. (1992). Dynamical theory of the laser-induced lattice instability of silicon. Phys. Rev. B.

[24] Hu A., Rybachuk M., Lu Q.-B., Duley W.W. (2007). Direct synthesis of sp-bonded carbon chains on graphite surface by femtosecond laser irradiation. Appl. Phys. Lett..

[25] Varel H., Wähmer M., Rosenfeld A., Ashkenasi D., Campbell E.E.B. (1998). Femtosecond laser ablation of sapphire: Time-of-flight analysis of ablation plume. Appl. Surf. Sci..

[26] Hu A. (2020). Laser Micro-Nano-Manufacturing and 3D Microprinting.

[27] Liu X., Du D., Mourou G. (1997). Laser ablation and micromachining with ultrashort laser pulses. IEEE J. Quantum Electron..

[28] Lin X., Chen H., Jiang S., Zhang C. (2012). A Coulomb explosion theoretical model of femtosecond laser ablation materials. Sci. China Technol. Sci..

[29] Jiang L., Wang A.-D., Li B., Cui T.-H., Lu Y.-F. (2018). Electrons dynamics control by shaping femtosecond laser pulses in micro/nanofabrication: Modeling, method, measurement and application. Light Sci. Appl..

[30] Liu H., Sun S., Zheng L., Wang G., Tian W., Zhang D., Han H., Zhu J., Wei Z. (2021). Review of laser-diode pumped Ti:sapphire laser. Microw. Opt. Technol. Lett..

[31] Cao X., Li Q., Li F., Zhao H., Zhao W., Wang Y., Li D., Yang Y., Wen W., Si J. (2023). Femtosecond Yb-doped tapered fiber pulse amplifiers with peak power of over hundred megawatts. Opt. Express.

[32] Lesparre F., Gomes J.T., Délen X., Martial I., Didierjean J., Pallmann W., Resan B., Eckerle M., Graf T., Ahmed M.A. (2015). High-power Yb:YAG single-crystal fiber amplifiers for femtosecond lasers in cylindrical polarization. Opt. Lett..

[33] Strickland D., Mourou G. (1985). Compression-of-amplified-chirped-optical-pulses. Opt. Commun..

[34] Maibohm C., Silvestre O.F., Borme J., Sinou M., Heggarty K., Nieder J.B. (2020). Multi-beam two-photon polymerization for fast large area 3D periodic structure fabrication for bioapplications. Sci. Rep..

[35] Nakata Y., Murakawa K., Miyanaga N., Narazaki A., Shoji T., Tsuboi Y. (2018). Local melting of gold thin films by femtosecond laser-interference processing to generate nanoparticles on a source target. Nanomaterials.

[36] Lin Y., Shi H., Jia T. (2021). Distortion and Light Intensity Correction for Spatiotemporal-Interference-Based Spatial Shaping. Laser Optoelectron. Prog..

[37] Wittenbecher L., Zigmantas D. (2019). Correction of Fabry-Pérot interference effects in phase and amplitude pulse shapers based on liquid crystal spatial light modulators. Opt. Express.

[38] Wang T.-W., Dong X.-Z., Jin F., Zhao Y.-Y., Liu X.-Y., Zheng M.-L., Duan X.-M. (2022). Consistent pattern printing of the gap structure in femtosecond laser DMD projection lithography. Opt. Express.

[39] Somers P., Liang Z., Johnson J.E., Boudouris B.W., Pan L., Xu X. (2021). Rapid, continuous projection multi-photon 3D printing enabled by spatiotemporal focusing of femtosecond pulses. Light Sci. Appl..

[40] Choi J., Kim H., Saha S.K. (2023). Rapid printing of metal nanostructures through projection-based two-photon reduction. Manuf. Lett..

[41] Satoshi Hasegawa Y.H., Nishida N. (2006). Holographic femtosecond laser processing with multiplexed phase Fresnel lenses. Opt. Lett..

[42] Nguyen H.D., Sedao X., Mauclair C., Bidron G., Faure N., Moreno E., Colombier J.-P., Stoian R. (2020). Non-Diffractive Bessel Beams for Ultrafast Laser Scanning Platform and Proof-Of-Concept Side-Wall Polishing of Additively Manufactured Parts. Micromachines.

[43] Pan D., Liu S., Li J., Ni J., Xin C., Ji S., Lao Z., Zhang C., Xu B., Li R. (2022). Rapid Fabrication of 3D Chiral Microstructures by Single Exposure of Interfered Femtosecond Vortex Beams and Capillary-Force-Assisted Self-Assembly. Adv. Funct. Mater..

[44] Hu Y., Feng W., Xue C., Lao Z., Ji S., Cai Z., Zhu W., Li J., Wu D., Chu J. (2020). Self-assembled micropillars fabricated by holographic femtosecond multi-foci beams forin situ trapping of microparticles. Opt. Lett..

[45] Wang C., Yang L., Hu Y., Rao S., Wang Y., Pan D., Ji S., Zhang C., Su Y., Zhu W. (2019). Femtosecond mathieu beams for rapid controllable fabrication of complex microcages and application in trapping microobjects. ACS Nano.

[46] Yang D., Liu L., Gong Q., Li Y. (2019). Rapid Two-Photon Polymerization of an Arbitrary 3D Microstructure with 3D Focal Field Engineering. Macromol. Rapid Commun..

[47] Yue Y., Erse J., Xinyu M., Chen X., Bowen L., Yanfeng L., Minglie H. (2023). High throughput direct writing of a mesoscale binary optical element by femtosecond long focal depth beams. Light Adv. Manuf..

[48] Kadoguchi N., Uesugi Y., Nagasako M., Kobayashi T., Kozawa Y., Sato S. (2023). Nanoprocessing of Self-Suspended Monolayer Graphene and Defect Formation by Femtosecond-Laser Irradiation. Nano Lett..

[49] Saha S.K., Wang D., Nguyen V.H., Chang Y., Oakdale J.S., Chen S.-C. (2019). Scalable submicrometer additive manufacturing. Science.

[50] Weiner A.M. (2011). Ultrafast optical pulse shaping: A tutorial review. Opt. Commun..

[51] Liu W., Hu J., Jiang L., Huang J., Lu J., Yin J., Qiu Z., Liu H., Li C., Wang S. (2021). Formation of laser-induced periodic surface nanometric concentric ring structures on silicon surfaces through single-spot irradiation with orthogonally polarized femtosecond laser double-pulse sequences. Nanophotonics.

[52] Zhou S., Ouzounov D., Li H., Bazarov I., Dunham B., Sinclair C., Wise F.W. (2007). Efficient temporal shaping of ultrashort pulses with birefringent crystals. Appl. Opt..

[53] Wu H., Jiao Y., Zhang C., Chen C., Yang L., Li J., Ni J., Zhang Y., Li C., Zhang Y. (2019). Large area metal micro-/nano-groove arrays with both structural color and anisotropic wetting fabricated by one-step focused laser interference lithography. Nanoscale.

[54] Nakata Y., Hayashi E., Tsubakimoto K., Miyanaga N., Narazaki A., Shoji T., Tsuboi Y. (2020). Nanodot array deposition via single shot laser interference pattern using laser-induced forward transfer. Int. J. Extreme Manuf..

[55] Li B., Jiang L., Li X., Lin Z., Huang L., Wang A., Han W., Wang Z., Lu Y. (2018). Flexible gray-scale surface patterning through spatiotemporal-interference-based femtosecond laser shaping. Adv. Opt. Mater..

[56] Zhao Z., Yang J. (2022). Hybrid Grating-Hole Nanostructures Produced by Spatiotemporal Modulation of Femtosecond Lasers: Implications for Near-Field Enhancement. ACS Appl. Nano Mater..

[57] Yuan Y., Zhang Z., Li X., Jiang L., Zhang X., Zuo P., Xu C., Ma L., Wang S., Zhao Y. (2022). Bottom-up scalable temporally-shaped femtosecond laser deposition of hierarchical porous carbon for ultrahigh-rate micro-supercapacitor. Sci. China Mater..

[58] Yuan Y., Li X., Jiang L., Liang M., Zhang X., Wu S., Wu J., Tian M., Zhao Y., Qu L. (2023). Laser maskless fast patterning for multitype microsupercapacitors. Nat. Commun..

[59] Sharif A., Farid N., McGlynn P., Wang M., Vijayaraghavan R.K., Jilani A., Leen G., McNally P.J., O’connor G.M. (2023). Ultrashort laser sintering of printed silver nanoparticles on thin, flexible, and porous substrates. J. Phys. D Appl. Phys..

[60] Huang H., Sivayoganathan M., Duley W.W., Zhou Y. (2015). High integrity interconnection of silver submicron/nanoparticles on silicon wafer by femtosecond laser irradiation. Nanotechnology.

[61] Jihun Noh D.K. (2020). Femtosecond laser sintering of silver nanoparticles for conductive thin-film fabrication. Appl. Phys. A.

[62] Liao J., Wang X., Zhou X., Kang H., Guo W., Peng P. (2021). Joining Process of Copper Nanoparticles with Femtosecond Laser Irradiation. Chin. J. Lasers.

[63] Huang Y., Xie X., Li M., Xu M., Long J. (2021). Copper circuits fabricated on flexible polymer substrates by a high repetition rate femtosecond laser-induced selective local reduction of copper oxide nanoparticles. Opt. Express.

[64] Mizue Mizoshiri K.Y. (2021). Cu Patterning Using Femtosecond Laser Reductive Sintering of CuO Nanoparticles under Inert Gas Injection. Materials.

[65] Wan Z., Streed E.W., Lobino M., Wang S., Sang R.T., Cole I.S., Thiel D.V., Li Q. (2018). Laser-Reduced Graphene: Synthesis, Properties, and Applications. Adv. Mater. Technol..

[66] Low M.J., Lee H., Lim C.H.J., Sandeep C.S., Murukeshan V.M., Kim S.-W., Kim Y.-J. (2020). Laser-induced reduced-graphene-oxide micro-optics patterned by femtosecond laser direct writing. Appl. Surf. Sci..

[67] Zhang Y., Guo L., Wei S., He Y., Xia H., Chen Q., Sun H.-B., Xiao F.-S. (2010). Direct imprinting of microcircuits on graphene oxides film by femtosecond laser reduction. Nano Today.

[68] Jiang S., Park C.-S., Lee W.-B., Lee S.-S. (2021). Photoreduction-Insensitive GO/rGO Patterning Based on Multistep Femtosecond Laser Writing for Implementing Fresnel Zone Plates. ACS Appl. Nano Mater..

[69] Arakane S., Mizoshiri M., Sakurai J., Hata S. (2017). Direct writing of three-dimensional Cu-based thermal flow sensors using femtosecond laser-induced reduction of CuO nanoparticles. J. Micromechanics Microengineering.

[70] He J., Wang S., Jiang L., Li X., Hong Q., Zhu W., Sun J., Zhang X., Xu Z. (2022). Femtosecond Laser One-Step Direct Writing Electrodes with Ag NPs-Graphite Carbon Composites for Electrochemical Sensing. Adv. Mater. Technol..

[71] Liao J., Guo W., Peng P. (2021). Direct laser writing of copper-graphene composites for flexible electronics. Opt. Lasers Eng..

[72] Peng P., Li L., He P., Zhu Y., Fu J., Huang Y., Guo W. (2019). One-step selective laser patterning of copper/graphene flexible electrodes. Nanotechnology.

[73] Mizoshiri M., Tanokuchi A. (2020). Direct writing of Cu-based micropatterns inside Cu_2_O nanosphere films using green femtosecond laser reductive sintering. Opt. Mater. Express.

[74] Ha N.P., Ohishi T., Mizoshiri M. (2021). Direct Writing of Cu Patterns on Polydimethylsiloxane Substrates Using Femtosecond Laser Pulse-Induced Reduction of Glyoxylic Acid Copper Complex. Micromachines.

[75] Nguyen C.M., Batista L.M.F., John M.G., Rodrigues C.J., Tibbetts K.M. (2021). Mechanism of Gold–Silver Alloy Nanoparticle Formation by Laser Coreduction of Gold and Silver Ions in Solution. J. Phys. Chem. B.

[76] Mizoshiri M., Yoshidomi K., Darkhanbaatar N., Khairullina E.M., Tumkin I.I. (2021). Effect of Substrates on Femtosecond Laser Pulse-Induced Reductive Sintering of Cobalt Oxide Nanoparticles. Nanomaterials.

[77] Fedotov S.S., Lipat’eva T.O., Lipat’ev A.S., Shakhgil’dyan G.Y., Lotarev S.V., Savinkov V.I., Sigaev V.N. (2023). Femtosecond Laser Welding of Glass and Sitall with Substantially Different Values of the LTEC. Glas. Ceram..

[78] Hu A., Peng P., Alarifi H., Zhang X.Y., Guo J.Y., Zhou Y., Duley W.W. (2012). Femtosecond laser welded nanostructures and plasmonic devices. J. Laser Appl..

[79] Yu Y., Joshi P., Bridges D., Fieser D., Hu A. (2023). Femtosecond Laser-Induced Nano-Joining of Volatile Tellurium Nanotube Memristor. Nanomaterials.

[80] Hong S., Lee H., Yeo J., Ko S.H. (2016). Digital selective laser methods for nanomaterials: From synthesis to processing. Nano Today.

[81] Yu Y., Bai S., Wang S., Hu A. (2018). Ultra-Short Pulsed Laser Manufacturing and Surface Processing of Microdevices. Engineering.

[82] Hu A., Li R., Bridges D., Zhou W., Bai S., Ma D., Peng P. (2016). Photonic nanomanufacturing of high performance energy devices on flexible substrates. J. Laser Appl..

[83] Yu Y., Deng Y., Al Hasan A., Bai Y., Li R.-Z., Deng S., Joshi P.C., Shin S., Hu A. (2020). Femtosecond laser-induced non-thermal welding for a single Cu nanowire glucose sensor. Nanoscale Adv..

[84] Lin L., Huo J., Peng P., Zou G., Liu L., Duley W.W., Zhou Y.N. (2020). Contact engineering of single core/shell SiC/SiO_2_ nanowire memory unit with high current tolerance using focused femtosecond laser irradiation. Nanoscale.

[85] Miyakoshi R., Morosawa F., Hayashi S., Terakawa M. Electrically conductive porous carbon structures fabricated by laser direct carbonization of bamboo. Proceedings of the 2021 Conference on Lasers and Electro-Optics Europe & European Quantum Electronics Conference (CLEO/Europe-EQEC).

[86] Kim Y.-J., Le T.-S.D., Nam H.K., Yang D., Kim B. (2021). Wood-based flexible graphene thermistor with an ultra-high sensitivity enabled by ultraviolet femtosecond laser pulses. CIRP Ann..

[87] Bin In J., Hsia B., Yoo J.-H., Hyun S., Carraro C., Maboudian R., Grigoropoulos C.P. (2015). Facile fabrication of flexible all solid-state micro-supercapacitor by direct laser writing of porous carbon in polyimide. Carbon.

[88] Morosawa F., Hayashi S., Terakawa M. (2021). Femtosecond Laser-Induced Graphitization of Transparent Cellulose Nanofiber Films. ACS Sustain. Chem. Eng..

[89] Morita N., Shimotsuma Y., Nishi M., Sakakura M., Miura K., Hirao K. (2014). Direct micro-carbonization inside polymer using focused femtosecond laser pulses. Appl. Phys. Lett..

[90] Wang S., Yu Y., Li R., Feng G., Wu Z., Compagnini G., Gulino A., Feng Z., Hu A. (2017). High-performance stacked in-plane supercapacitors and supercapacitor array fabricated by femtosecond laser 3D direct writing on polyimide sheets. Electrochimica Acta.

[91] Wang S., Yu Y., Luo S., Cheng X., Feng G., Zhang Y., Wu Z., Compagnini G., Pooran J., Hu A. (2019). All-solid-state supercapacitors from natural lignin-based composite film by laser direct writing. Appl. Phys. Lett..

[92] Bai R., Gao Y., Lu C., Tan J., Xuan F. (2021). Femtosecond laser micro-fabricated flexible sensor arrays for simultaneous mechanical and thermal stimuli detection. Measurement.

[93] Hong Q., Zhu W., Wang S., Jiang L., He J., Zhan J., Li X., Zhao X., Zhao B. (2022). High-Resolution Femtosecond Laser-Induced Carbon and Ag Hybrid Structure for Bend Sensing. ACS Omega.

[94] Benzigar M.R., Dasireddy V.D.B.C., Guan X., Wu T., Liu G. (2020). Advances on Emerging Materials for Flexible Supercapacitors: Current Trends and Beyond. Adv. Funct. Mater..

[95] Yasami S., Mazinani S., Abdouss M. (2023). Developed composites materials for flexible supercapacitors electrode: “Recent progress & future aspects”. J. Energy Storage.

[96] Mohan M., Shetti N.P., Aminabhavi T.M. (2023). Recent developments in MoS2-based flexible supercapacitors. Mater. Today Chem..

[97] Rani S., Kumar N., Sharma Y. (2021). Recent progress and future perspectives for the development of micro-supercapacitors for portable/wearable electronics applications. J. Physics: Energy.

[98] Sun H., Zhu J., Baumann D., Peng L., Xu Y., Shakir I., Huang Y., Duan X. (2019). Hierarchical 3D electrodes for electrochemical energy storage. Nat. Rev. Mater..

[99] Lai W., Wang Y., Wang X., Nairan A., Yang C. (2018). Fabrication and Engineering of Nanostructured Supercapacitor Electrodes Using Electromagnetic Field-Based Techniques. Adv. Mater. Technol..

[100] Kwon S., Jung D., Lim H., Kim G., Choi K.-B., Lee J. (2017). Laser-assisted selective lithography of reduced graphene oxide for fabrication of graphene-based out-of-plane tandem microsupercapacitors with large capacitance. Appl. Phys. Lett..

[101] Keum K., Kim J.W., Hong S.Y., Son J.G., Lee S., Ha J.S. (2020). Flexible/Stretchable Supercapacitors with Novel Functionality for Wearable Electronics. Adv. Mater..

[102] Imbrogno A., Islam J., Santillo C., Castaldo R., Sygellou L., Larrigy C., Murray R., Vaughan E., Hoque K., Quinn A.J. (2022). Laser-Induced Graphene Supercapacitors by Direct Laser Writing of Cork Natural Substrates. ACS Appl. Electron. Mater..

[103] Aeby X., Poulin A., Siqueira G., Hausmann M.K., Nyström G. (2021). Fully 3D Printed and Disposable Paper Supercapacitors. Adv. Mater..

[104] Huang F., Zhou S., Yan Z., Wang S., Zhang H., Wang S., Zhou S. (2023). Laser carbonization of lignin-based fiber membranes with heating treatment for flexible supercapacitors. Appl. Surf. Sci..

[105] Li R.-Z., Peng R., Kihm K.D., Bai S., Bridges D., Tumuluri U., Wu Z., Zhang T., Compagnini G., Feng Z. (2016). High-rate in-plane micro-supercapacitors scribed onto photo paper using in situ femtolaser-reduced graphene oxide/Au nanoparticle microelectrodes. Energy Environ. Sci..

[106] Le T.D., Lee Y.A., Nam H.K., Jang K.Y., Yang D., Kim B., Yim K., Kim S., Yoon H., Kim Y. (2022). Green Flexible Graphene–Inorganic-Hybrid Micro-Supercapacitors Made of Fallen Leaves Enabled by Ultrafast Laser Pulses. Adv. Funct. Mater..

[107] Kumar R., del Pino A.P., Sahoo S., Singh R.K., Tan W.K., Kar K.K., Matsuda A., Joanni E. (2022). Laser processing of graphene and related materials for energy storage: State of the art and future prospects. Prog. Energy Combust. Sci..

[108] Yuan Y., Jiang L., Li X., Zuo P., Zhang X., Lian Y., Ma Y., Liang M., Zhao Y., Qu L. (2022). Ultrafast Shaped Laser Induced Synthesis of MXene Quantum Dots/Graphene for Transparent Supercapacitors. Adv. Mater..

[109] Luo J., Gao W., Wang Z.L. (2021). The Triboelectric Nanogenerator as an Innovative Technology toward Intelligent Sports. Adv. Mater..

[110] Khandelwal G., Raj N.P.M.J., Kim S.-J. (2020). Triboelectric nanogenerator for healthcare and biomedical applications. Nano Today.

[111] Kim D., Tcho I.-W., Jin I.K., Park S.-J., Jeon S.-B., Kim W.-G., Cho H.-S., Lee H.-S., Jeoung S.C., Choi Y.-K. (2017). Direct-laser-patterned friction layer for the output enhancement of a triboelectric nanogenerator. Nano Energy.

[112] Huang J., Fu X., Liu G., Xu S., Li X., Zhang C., Jiang L. (2019). Micro/nano-structures-enhanced triboelectric nanogenerators by femtosecond laser direct writing. Nano Energy.

[113] Zhang H., Yin K., Wang L., Deng Q., He Y., Xiao Z., Li G., Dai G. (2023). A Robust Droplet Triboelectric Nanogenerator with Self-Cleaning Ability Achieved by Femtosecond Laser. ACS Appl. Mater. Interfaces.

[114] Khalid M.A.U., Chang S.H. (2022). Flexible strain sensors for wearable applications fabricated using novel functional nanocomposites: A review. Compos. Struct..

[115] Chen H., Zhuo F., Zhou J., Liu Y., Zhang J., Dong S., Liu X., Elmarakbi A., Duan H., Fu Y. (2023). Advances in graphene-based flexible and wearable strain sensors. Chem. Eng. J..

[116] Yan Z., Wang S., Huang F., Deng G., Sui X., Wu Z., Wang J. (2023). High sensitivity iontronic pressure sensors with wavy structure electrode and two-level raised structures ionic gel film prepared by direct laser writing. Sens. Actuators A Phys..

[117] Shen L., Zhou S., Gu B., Wang S., Wang S. (2023). Highly Sensitive Strain Sensor Fabricated by Direct Laser Writing on Lignin Paper with Strain Engineering. Adv. Eng. Mater..

[118] An J., Le T.-S.D., Huang Y., Zhan Z., Li Y., Zheng L., Huang W., Sun G., Kim Y.-J. (2017). All-Graphene-Based Highly Flexible Noncontact Electronic Skin. ACS Appl. Mater. Interfaces.

[119] Wang Y.-L., Li B.-J., Li S.-S., Huang L.-J., Wang Y.-Y., Ren N.-F. (2021). Fabrication of metal mesh flexible transparent electrodes and heaters by a cost-effective method based on ultrafast laser direct writing. Opt. Laser Technol..

[120] Ji Y., Liao Y., Li H., Cai Y., Fan D., Liu Q., Huang S., Zhu R., Wang S., Wang H. (2022). Flexible Metal Electrodes by Femtosecond Laser-Activated Deposition for Human–Machine Interfaces. ACS Appl. Mater. Interfaces.

[121] Yang D., Nam H.K., Le T.-S.D., Yeo J., Lee Y., Kim Y.-R., Kim S.-W., Choi H.-J., Shim H.C., Ryu S. (2023). Multimodal E-Textile Enabled by One-Step Maskless Patterning of Femtosecond-Laser-Induced Graphene on Nonwoven, Knit, and Woven Textiles. ACS Nano.

[122] Yi P., Fu X., Liu Y., Zhang X., Zhang C., Li X. (2023). Triboelectric active pressure sensor with ultrabroad linearity range by femtosecond laser shaping based on electrons dynamics control. Nano Energy.

[123] Zhu W., Wang M., Zhang Z., Sun J., Zhan J., Guan M., Xu Z., Wang S., Li X., Jiang L. (2023). Controllable Photoreduction of Graphene Oxide/Gold Composite Using a Shaped Femtosecond Laser for Multifunctional Sensors. ACS Appl. Mater. Interfaces.

[124] Du H., Zhang N., Xiong B., Zhang X., Yuan X. (2023). High performance flexible PVDF film pressure sensor fabricated by femtosecond laser. Opt. Laser Technol..

[125] Chen L., Hu Y., Huang H., Liu C., Zang Y., Wu D., Xia J. (2022). Femtosecond Laser-Assisted Device Engineering: Toward Organic Field-Effect Transistor-Based High-Performance Gas Sensors. ACS Appl. Mater. Interfaces.

[126] Park H., Kim J.-H., Shin W.-S., Mirzaei A., Kim Y.-J., Kim S.S., Halik M., Park C. (2022). Facile strategy for advanced selectivity and sensitivity of SnO2 nanowire-based gas sensor using chemical affinity and femtosecond laser irradiation. Sens. Actuators B Chem..

[127] Kim J.-H., Mirzaei A., Kim S.S., Park C. (2023). Pt nanoparticle decoration on femtosecond laser-irradiated SnO2 nanowires for enhancing C7H8 gas sensing. Sens. Actuators B Chem..

[128] Nam H.K., Le T.S.D., Yang D., Kim B., Lee Y., Hwang J.S., Kim Y.R., Yoon H., Kim S.W., Kim Y.J. (2023). Smart Wooden Home Enabled by Direct-Written Laser-Induced Graphene. Adv. Mater. Technol..

[129] Sharif A., Farid N., Collins A., Jilani A., O’Connor G.M. (2023). Extensive reduction of graphene oxide on thin polymer substrates by ultrafast laser for robust flexible sensor applications. Appl. Surf. Sci..

[130] An S., Park H., Kim M. (2023). Recent advances in single crystal narrow band-gap semiconductor nanomembranes and their flexible optoelectronic device applications: Ge, GeSn, InGaAs, and 2D materials. J. Mater. Chem. C.

[131] Righini G.C., Krzak J., Lukowiak A., Macrelli G., Varas S., Ferrari M. (2021). From flexible electronics to flexible photonics: A brief overview. Opt. Mater..

[132] An J., Le T.D., Lim C.H.J., Tran V.T., Zhan Z., Gao Y., Zheng L., Sun G., Kim Y. (2018). Single-Step Selective Laser Writing of Flexible Photodetectors for Wearable Optoelectronics. Adv. Sci..

[133] Wang Y., Liu W., Xin W., Zou T., Zheng X., Li Y., Xie X., Sun X., Yu W., Liu Z. (2020). Back-Reflected Performance-Enhanced Flexible Perovskite Photodetectors through Substrate Texturing with Femtosecond Laser. ACS Appl. Mater. Interfaces.

[134] Liang S., Dai Y., Wang G., Xia H., Zhao J. (2020). Room-temperature fabrication of SiC microwire photodetectors on rigid and flexible substrates via femtosecond laser direct writing. Nanoscale.

[135] Lin H., Sturmberg B.C.P., Lin K.-T., Yang Y., Zheng X., Chong T.K., de Sterke C.M., Jia B. (2019). A 90-nm-thick graphene metamaterial for strong and extremely broadband absorption of unpolarized light. Nat. Photon..

[136] Zou T., Zhao B., Xin W., Wang Y., Wang B., Zheng X., Xie H., Zhang Z., Yang J., Guo C.-L. (2020). High-speed femtosecond laser plasmonic lithography and reduction of graphene oxide for anisotropic photoresponse. Light Sci. Appl..

[137] Zou T., Zhao B., Xin W., Wang F., Xie H., Li Y., Shan Y., Li K., Sun Y., Yang J. (2021). Birefringent response of graphene oxide film structurized via femtosecond laser. Nano Res..

[138] Heffner H., Soldera M., Ränke F., Lasagni A.F. (2023). Surface Modification of Fluorine-Doped Tin Oxide Thin Films Using Femtosecond Direct Laser Interference Patterning: A Study of the Optoelectronic Performance. Adv. Eng. Mater..

